# Human liver organoids generated with single donor-derived multiple cells rescue mice from acute liver failure

**DOI:** 10.1186/s13287-017-0749-1

**Published:** 2018-01-10

**Authors:** Yun-Zhong Nie, Yun-Wen Zheng, Miyuki Ogawa, Etsuko Miyagi, Hideki Taniguchi

**Affiliations:** 1 Department of Regenerative Medicine, Yokohama City University Graduate School of Medicine, Yokohama, Kanagawa 236-0004 Japan; 2 Department of Advanced Gastroenterological Surgical Science and Technology, Faculty of Medicine, University of Tsukuba, Tsukuba-shi, Ibaraki 305-8575 Japan; 3 Research Center of Stem Cells and Regenerative Medicine, Jiangsu University Hospital, Zhenjiang, Jiangsu 212001 China; 4 Department of Obstetrics and Gynecology, Yokohama City University Graduate School of Medicine, Yokohama, Kanagawa 236-0004 Japan; 5 Advanced Medical Research Center, Yokohama City University Graduate School of Medicine, Yokohama, Kanagawa 236-0004 Japan

**Keywords:** Liver organoids, Single donor, Human induced pluripotent stem cells, Acute liver failure, Liver regeneration

## Abstract

**Background:**

Acute liver failure (ALF) is a life-threatening disease with a high mortality rate. However, there are limited treatments or devices available for ALF therapy. Here, we aimed to develop a new strategy for ALF treatment by transplanting functional liver organoids (LOs) generated from single donor-derived human induced pluripotent stem cell (hiPSC) endoderm, endothelial cells (ECs), and mesenchymal cells (MCs).

**Methods:**

First, we isolated ECs and MCs from a single donor umbilical cord (UC) through enzyme digestion and characterized the UC-ECs and UC-MCs by flow cytometry. Second, using a nonviral reprogramming method, we generated same donor-derived hiPSCs from the UC-ECs and investigated their hepatic differentiation abilities. Finally, we simultaneously plated EC-hiPSC endoderm, UC-ECs, and UC-MCs in a three-dimensional (3D) microwell culture system, and generated single donor cell-derived differentiated LOs for ALF mouse treatment.

**Results:**

We obtained ECs and MCs from a single donor UC with high purity, and these cells provided a multicellular microenvironment that promoted LO differentiation. hiPSCs from the same donor were generated from UC-ECs, and the resultant EC-hiPSCs could be differentiated efficiently into pure definitive endoderm and further into hepatic lineages. Simultaneous plating of EC-hiPSC endoderm, UC-ECs, and UC-MCs in the 3D microwell system generated single donor cell-derived LOs (SDC-LOs) that could be differentiated into functional LOs with enhanced hepatic capacity as compared to that of EC-hiPSC-derived hepatic-like cells. When these functional SDC-LOs were transplanted into the renal subcapsules of ALF mice, they rapidly assumed hepatic functions and improved the survival rate of ALF mice.

**Conclusion:**

These results demonstrate that functional LOs generated from single donor cells can improve the condition of ALF mice. Functional SDC-LO transplantation provides a promising novel approach for ALF therapy.

**Electronic supplementary material:**

The online version of this article (doi:10.1186/s13287-017-0749-1) contains supplementary material, which is available to authorized users.

## Background

Acute liver failure (ALF) is a rare but life-threatening illness that is mainly caused by viral infection and drug-induced liver injury [1]. The clinical presentation of ALF initially includes hepatic dysfunction, abnormal liver biochemical values, and coagulopathy, followed by the development of encephalopathy with multiple organ failure resulting in mortality in 80–85% of cases [1]. Although various treatments are administered to prevent disease progression in these patients, the preferred option is liver transplantation, which has 1-year and 5-year survival rates of 79% and 72%, respectively [2]. However, transplantation is not universally available (performed in < 10% of patients with ALF [2, 3]), and an increased risk of death is associated with transplantation with infected, older, or partial grafts or grafts from donors without matching human leukocyte antigen (HLA) [2, 4].

As an alternative to liver transplantation, primary human hepatocyte (PHH) transplantation has been performed in patients with liver-based metabolic disease and ALF, and in neonates and children with metabolic disorders [5]. However, this treatment is also limited by donor shortage [6]. This shortage in PHHs might be resolved with the emergence of human induced pluripotent stem cells (hiPSCs), which could provide an unlimited source of hepatic-like cells (HLCs) [7, 8]. hiPSC-HLCs have been used for engraftment in a liver injury model and to repair injured liver tissue [9]; however, following engraftment, it took weeks for hiPSC-HLCs to start producing detectable levels of specific proteins and exhibit hepatic function [10]. Because of the rapid deterioration of liver function and extensive toxic substance accumulation [1], the treatment window for ALF patients is very narrow, and the extended time period needed for engraftment with hiPSC-HLCs might not be suitable for ALF therapy. Therefore, transplants that can assume hepatic functions with little delay are required for ALF treatment.

Transplantation of in vitro-generated organoids can regenerate functional tissue in vivo and might be a potential approach for ALF treatment [11]. Previous studies have developed a macro-liver organoid (macro-LO) from hiPSCs with endothelial cells (ECs) and mesenchymal cells (MCs) that can grow into vascularized, functional tissue post transplantation by recapitulating events that occur in early liver organogenesis [12, 13]. However, the three types of cells necessary for LO generation were derived from three different donors with different HLA types, and thus would not match any particular patient’s HLA for clinical application. Clinical liver transplantation studies have found that incompatible HLA matching between the graft and the patient is associated with increased mortality and graft loss in ALF patient therapy [2]; therefore, LOs derived from a single donor cell should be developed to match specific patients’ HLAs. Single donor-derived cells have the same host genetic background and thus may recapitulate liver organogenesis more accurately to support LO differentiation. Additionally, the LOs reported previously were still at an immature stage, and they required 2 weeks to differentiate and exhibit hepatic function with human-specific albumin (ALB) production in vivo [12]. However, for ALF therapy, transplants that can immediately exhibit hepatic function in vivo are urgently needed [14], and functional LOs are thus considered to hold promise. Therefore, functional LOs generated from single donor cells are expected to be useful for ALF therapy.

To generate functional single donor cell-derived (SDC)-LOs for clinical application, all three types of cells (hiPSC-endoderm, ECs, and MCs) should be obtained from the same donor with high purity and few genomic mutations. Human somatic cells accumulate nuclear and mitochondrial genomic mutations over the course of their lifespan [15]; thus, cells obtained from newborns would be expected to have fewer mutations than adult cells. ECs and MCs that have been used for LO generation are derived from newborn umbilical cords (UCs) and adult bone marrow (BM), respectively [12, 13]. The isolation of MCs from newborn BM requires an invasive procedure that would cause harm to the newborn, so a replacement MC source from the same newborn is thus necessary for SDC-LO generation. The newborn UC is an immature tissue with a large number of MCs that can be isolated without invasive procedures [16], and UC-derived MCs have characteristics similar to those of BM-derived MCs (BM-MCs) [16]. Therefore, ECs and MCs derived from the same UC might be useful for generating functional SDC-LOs with the same donor-derived hiPSCs.

In this study, we obtained ECs and MCs from a single donor UC and investigated the role of single donor-derived ECs and MCs in LO generation and differentiation. We also generated hiPSCs from the same donor to provide a hiPSC-endoderm source for LO generation. Using these three types of cells, we generated functional SDC-LOs for ALF therapy.

## Methods

### EC and MC isolation and culture

Under the permission of the Ethical Review Board of Yokohama City University, human UCs were obtained from the Department of Obstetrics and Gynecology, Yokohama City University Hospital from full-term caesarian section births after obtaining informed consent from the mother. ECs and MCs were collected from the same UC. The collection procedure was in accordance with the ethical standards of the local ethics committee. To isolate ECs [17], the cord was laid out on a clean disk; the cord vein was washed three times with phosphate-buffered saline (PBS) and filled with 10 ml collagenase (1 mg/ml; Sigma-Aldrich, St. Louis, MO, USA). After clamping of the open end, the cord was incubated in RPMI 1640 medium at 37 °C for 15 min. The vein was washed with RPMI 1640 medium, the wash medium was collected and centrifuged at 200 × *g* for 5 min, and the pellet was resuspended in 10 ml EC growth medium (EGM; Lonza, Walkersville, MD, USA) and seeded in a 10-cm 0.1% gelatin-coated dish. For MC isolation, the vein and arteries were removed from the cord after isolation of ECs, and the cord was cut into pieces 1–2 mm^3^ that were incubated in an enzyme cocktail that included 2500 U/ml collagenase (Gibco, Grand Island, NY, USA), 5 mg/ml hyaluronidase (Wako Pure Chemical Industries, Osaka, Japan), and 2 U/ml dispase (Roche Diagnostics, Indianapolis, IN, USA) for 4 h with light shaking at 37 °C. After incubation, the sample was centrifuged at 400 × *g* for 10 min; the pellet was washed once with RPMI 1640 and resuspended in 10 ml mesenchymal stem cell growth medium (MSCGM; Lonza), and cells were seeded in a 10-cm tissue culture dish. ECs and BM-derived mesenchymal stem cells were obtained from Lonza as control ECs (con-ECs) and MCs (con-MCs) and maintained in EGM and MSCGM, respectively. All cells were maintained at 37 °C in a humidified incubator with 5% CO_2_.

### Generation of nonviral feeder-free hiPSCs from UC-derived ECs

Feeder-free hiPSCs were reprogrammed from ECs using a protocol reported previously [18], with minor modifications. Briefly, ECs were transfected with episomal iPSC reprogramming vectors (pCXLE-hOCT3/4-shp53-F, pCXLE-hSK, pCXLE-hUL, and pCXWB-EBNA1) using Nucleofector 4D and then cultured in a plate coated with growth factor-reduced Matrigel (BD Biosciences, Franklin Lakes, NJ, USA) in mTeSR medium (Stem Cell Technologies, Vancouver, BC, Canada). When the size of hiPSC colonies exceeded 1 mm, the colonies were picked and cultured in a plate coated with growth factor-reduced Matrigel in mTeSR medium to establish individual hiPSC lines. The TkDA3 human iPSC clone used in this study was provided by K. Eto and H. Nakauchi, University of Tokyo. Undifferentiated iPSCs were maintained in mTeSR1 medium on a dish coated with growth factor-reduced Matrigel. All cells were maintained at 37 °C in a humidified incubator with 5% CO_2_.

### Hepatic lineage differentiation and LO differentiation

HLCs were differentiated from hiPSCs according to a published protocol [7], with minor modifications.

To generate hiPSC-LOs, hiPSC endoderm cells (250,000 cells), con-ECs (175,000 cells), and con-MCs (25,000 cells) or UC-derived ECs (UC-EC) (175,000 cells) and MCs (UC-MC) (25,000 cells) were cocultured in serum-free differentiation (SFD) medium containing epidermal growth factor (EGF, 10 ng/ml; Sigma-Aldrich), vascular endothelial growth factor (VEGF, 10 ng/ml; Life Technologies, Carlsbad, CA, USA), basic fibroblast growth factor (bFGF, 10 ng/ml; Wako Pure Chemical Industries), hepatocyte growth factor (HGF, 20 ng/ml; Sigma-Aldrich), and dexamethasone (100 nM; Sigma-Aldrich) in a three-dimensional (3D) microwell plate (Kuraray, Tokyo, Japan). The SFD medium contained 375 ml Iscove’s modified Dulbecco’s medium (Life Technologies), 125 ml Ham’s F-12 K medium (Life Technologies), 5 ml B27 supplement (Life Technologies), 2.5 ml N2 supplement (Life Technologies), 0.05% bovine serum albumin (Sigma-Aldrich), 2 mM l-glutamine (Life Technologies), 1% penicillin–streptomycin (Life Technologies), 0.45 mM monothioglycerol solution (Wako Pure Chemical Industries), and 0.5 mM l-ascorbic acid (Sigma-Aldrich). The hepatic lineage cells and LOs were differentiated and maintained at 37 °C in a humidified incubator with 5% CO_2_.

### Macro-LO generation

Macro-LOs were generated from hiPSCs as described previously with minor modifications [19]. To generate macro-LOs, hiPSC endoderm (500,000 cells), con-ECs (350,000 cells), and con-MCs (50,000 cells) or UC-ECs (350,000 cells) and UC-MCs (50,000 cells) were resuspended in SFD medium containing EGF (10 ng/ml), VEGF (10 ng/ml), bFGF (10 ng/ml), HGF (20 ng/ml), and dexamethasone (100 nM) and were plated on presolidified growth factor-reduced Matrigel diluted with SFD medium (100 μl Matrigel + 100 μl SFD medium, incubated at 37 °C for at least 30 min to solidify) in a 48-well plate. Images of macro-LOs were taken at 0, 3, 12, 24, 48, and 72 h during formation. The macro-LO area and culture well area at each time point were quantified using ImageJ software (WS Rasband, ImageJ; NIH, Bethesda, MD, USA) and the following equation:

Percent area of LO = (LO area) / (Culture well area) × 100%.

The generated macro-LOs were cultured at 37 °C in a humidified incubator with 5% CO_2_.

### Primary human hepatocyte culture

The dish-plated freshly isolated PHHs from humanized mice were purchased from PhoenixBio Co., Ltd (Higashihiroshima, Japan), without cryopreservation. The PHHs were cultured in hepatic growth medium (PhoenixBio). After 24 h of culture, PHHs were used for ALB and urea production analysis.

### Transplantation of SDC-LOs into ALF mice

Alb-TRECK/SCID mice were a gift from the Tokyo Metropolitan Institute of Medical Science. The mice were bred and maintained according to the Yokohama City University institutional guidelines for the use of laboratory animals. All experimental procedures were approved by the institutional review board of the Animal Research Center, Yokohama City University School of Medicine (No. 075). The ALF model was generated as reported previously [20]. Briefly, 8–10-week-old mice were administered 1.5 μg/kg diphtheria toxin (DT) by intraperitoneal injection; 48 h later, serum alanine aminotransferase (ALT) and aspartate aminotransferase (AST) were detected using DRI-CHEM (Fujifilm, Tokyo, Japan) according to the manufacturer’s instructions. In vitro-generated and differentiated SDC-LOs (~1 × 10^6^ hepatocytes) were collected and transplanted into the renal subcapsular space of each ALF mouse. The sham group received 50 μl of sterile saline.

### RNA isolation, cDNA synthesis, and quantitative polymerase chain reaction

Total RNA was isolated using a PureLink viral RNA mini kit (Thermo Fisher Scientific, Waltham, MA, USA). Single-stranded cDNA was synthesized from RNA (1 μg) using a high-capacity cDNA reverse transcription kit (Thermo Fisher Scientific) according to the manufacturer’s instructions and was used for quantitative polymerase chain reaction (qPCR) with the specific primers and universal probe library probes presented in Additional file 1: Table S1. Target gene expression levels were calculated by the ΔΔCT method, with β-ACTIN serving as an internal control for normalization.

### Flow cytometry

Cells were labeled with antibodies against cluster of differentiation (CD)31, CD146, CD144, CD90, CD45, CD73, CD105, HLA-DR, SSEA4, and TRA-1-60 (BD Biosciences) and analyzed by flow cytometry on a MoFlo Astrios system (Beckman Coulter, Fullerton, CA, USA).

### ALB assay, cytochrome P450 3A4 assay, urea assay, and cell normalization

Human ALB levels were measured by enzyme-linked immunosorbent assay (ELISA) using a kit (Bethyl Laboratories, Montgomery, TX, USA). Urea production was evaluated using a QuantiChrom urea assay kit (BioAssay Systems, Hayward, CA, USA), and cytochrome P450 (CYP)3A4 activity was detected using a P450-Glo CYP3A4 assay kit (Promega, Madison, WI, USA) according to the manufacturer’s instructions.

The total cell numbers in hiPSC-HLCs and hiPSC-LOs were normalized according to DNA amount. To calculate the cell number in LOs, we first counted the number of hiPSC-HLCs using an IN Cell Analyzer 2000 (GE Healthcare, Cardiff, UK) with Hoechst 33342 staining. Second, total DNA of hiPSC-LOs and hiPSC-HLCs was extracted using DNeasy Blood & Tissue Kit (Qiagen, Hilden, Germany), and DNA was eluted in 50 μl of elusion buffer. Third, DNA concentration was determined using a nanodrop spectrophotometer (Thermo Fischer Scientific, Waltham, MA, USA). The total cell number in hiPSC-LOs was calculated using the following equation:

$$ \mathrm{hiPSC}\hbox{-} \mathrm{LO}\kern0.5em \mathrm{cell}\kern0.5em \mathrm{number}\kern0.5em =\kern0.5em \frac{\left(\mathrm{HLC}\kern0.5em \mathrm{cell}\kern0.5em \mathrm{number}\right)\kern0.5em \times \kern0.5em \left(\mathrm{LO}\kern0.5em \mathrm{DNA}\kern0.5em \mathrm{amount}\right)}{\left(\mathrm{HLC}\kern0.5em \mathrm{DNA}\kern0.5em \mathrm{amount}\right)} $$

ALB secretion, urea production, and CYP3A4 activity were also normalized to the calculated total cell number.

### Periodic acid–Schiff staining and indocyanine green uptake and release

Glycogen was detected with the periodic acid–Schiff (PAS) staining kit (Muto Pure Chemicals, Tokyo, Japan) according to the manufacturer’s instructions. Cells were washed with PBS and fixed in 4% paraformaldehyde for 15 min at room temperature. After washing with PBS, the cells were oxidized in 0.5% periodic acid solution for 7 min, then washed with PBS and incubated in Schiff reagent for 15 min. After three rounds of incubation for 2 min each in sulfurous acid water, the cells were washed with PBS and visualized by microscopy.

Dry indocyanine green (ICG) powder (Akorn, Buffalo Grove, IL, USA) (10 mg) was dissolved in 10 ml of hepatocyte culture medium (Lonza) to obtain a 1 mg/ml stock solution. The cells were cultured in ICG medium for 4 h at 37 °C, washed three times with PBS, and incubated in fresh medium for 4 h to determine ICG release.

### Immunohistochemistry

SDC-LOs and kidney tissue transplanted with SDC-LOs were embedded in optimal cutting temperature compound (Sakura Finetek, Tokyo, Japan), and 7-μm sections were cut and mounted on MAS-GP type A-coated slides (Matsunami, Osaka, Japan). Tissue sections and cultured cells were fixed in a 4% paraformaldehyde solution in PBS for 10 min, washed three times with PBS, and blocked for 60 min with 10% enhanced chemiluminescence prime blocking agent in PBS containing 0.3% Triton X-100, followed by three washes with PBS. Samples were incubated overnight at 4 °C with antibodies against human ALB (Bethyl Laboratories), Hepatic nuclear factor (HNF)4A (Santa Cruz Biotechnology, Santa Cruz, CA, USA) and α1-antitrypsin (A1AT) (Wako) in blocking buffer, washed three times with PBS, and incubated with a fluorophore-conjugated secondary antibody for 60 min at room temperature. The samples were washed three times in PBS and covered with mounting medium containing 4′,6-diamidino-2-phenylindole. Fluorescence was detected on an Axio Imager M1 microscope (Zeiss, Oberkochen, Germany).

### Statistical analysis

Data are expressed as the mean ± standard error. The means of two groups were compared by the Mann–Whitney *U* test or unpaired *t* test. *P* < 0.05 was considered to indicate statistically significant differences. Data were obtained from at least three independent biological replicates, and analyses were performed with GraphPad Prism software (San Diego, CA, USA).

## Results

### Single donor-derived UC-ECs and UC-MCs promote hiPSC-LO differentiation

To generate SDC-LOs for ALF therapy, we first isolated UC-ECs and UC-MCs from a single UC by digestion with an enzyme or an enzyme cocktail (Additional file 2: Figure S1A, B). UC-ECs demonstrated the same capacity as con-ECs for vascular network formation and the same proliferation rate (Additional file 2: Figure S1C, D). Flow cytometry analysis revealed that UC-ECs and con-ECs displayed similar surface markers: CD31^+^, CD144^+^, CD146^+^, CD90^−^, CD45^−^, and SSEA4^−^. However, the purity of UC-ECs was much higher than that of con-ECs (Additional file 2: Figure S1F). Moreover, we also analyzed and compared the characteristics of UC-MCs and con-MCs. The proliferation assay showed that UC-MCs had a higher proliferative capacity than con-MCs (Additional file 2: Figure S1E). Flow cytometry analysis revealed that UC-MCs and con-MCs were positive for CD90, CD73, and CD105, and negative for CD31, CD45, and HLA-DR. The purity of UC-MCs was also higher than that of con-MCs (Additional file 2: Figure S1G).

**Fig. 1:**
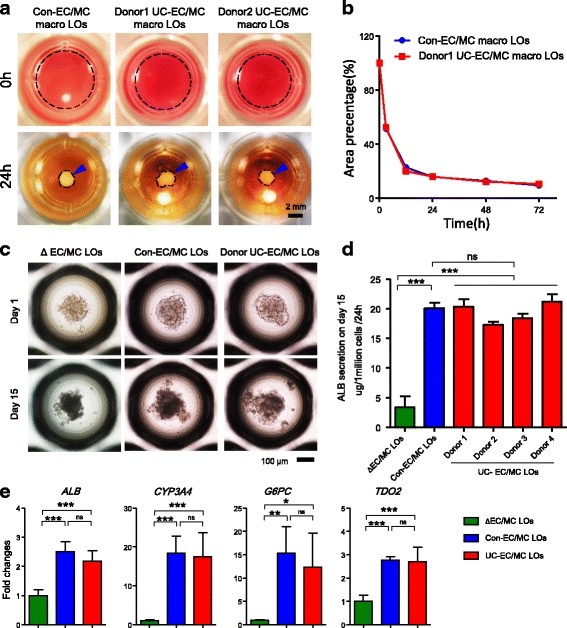
Single donor-derived UC-ECs and UC-MCs promote spontaneous LO formation and differentiation. **a** Macroscopic view of spontaneously generated macro-LOs in coculture with human iPSC-endoderm, control ECs, and BM-MCs (con-EC/MC) or single-donor UC-derived ECs and MCs (donor UC-EC/MC, two donors) at 0 and 24 h. Dotted line represents the organoid area. Blue arrowhead, macro-LOs. Scale bar, 2 mm. **b** Percent areas of macro-LOs during spontaneous formation from 0 to 72 h. **c** Morphology of ΔEC/MC LOs (deletion of ECs and MCs in LOs), con-EC/MC LOs (containing control ECs and MCs in LOs), and donor UC-EC/MC LOs (containing donor UC-ECs and UC-MCs in LOs) on days 1 and 15. Scale bar, 100 μm. **d** ALB production capacities of ΔEC/MC LOs (*n* = 4), con-EC/MC LOs (*n* = 6), and donor UC-EC/MC LOs (four donors, *n* = 3 each) on day 15. **e** *ALB*, *CYP3A4*, *G6PC*, and *TDO2* expression in ΔEC/MC LOs (green, *n* = 4), con-EC/MC LOs (blue, *n* = 6), and donor UC-EC/MC LOs (red, four donors, *n* = 12) on day 15, as determined by qPCR. *p < 0.05, **p < 0.01, ***p < 0.001. ALB albumin, ns not significant, Con control, EC endothelial cell, LO liver organoid, MC mesenchymal cell, UC umbilical cord, CYP cytochrome P450, G6PC glucose-6-phosphatase catalytic subunit, TDO2 tryptophan 2,3-dioxygenase

ECs and MCs have demonstrated the capacity to drive self-organization of hiPSC-endoderm into macro-LOs on a soft matrix [11, 12]. To investigate whether single donor-derived UC-ECs and UC-MCs could also drive this process, these cells along with hiPSC-endoderm were plated on the same soft matrix (Fig. 1a). We found that single donor-derived UC-ECs and UC-MCs (donor UC-EC/MC) could promote hiPSC-endoderm to spontaneously organize into 3D macro-LOs with a contraction speed equal to that of macro-LOs derived from con-ECs and con-MCs (con-EC/MC) used in previous studies (Fig. 1a, b) [11, 12].

The diameters of the self-organized macro-LOs were approximately 2 mm, and this size impeded their further differentiation in vitro. To promote LO differentiation and generate functional transplants, we used a 3D microwell culture system to generate small LOs by recreating the interactions among endoderm, con-ECs, and con-MCs (con-EC/MC LOs) (Fig. 1c). After 24 h, the three types of cells organized into con-EC/MC LOs with a small and uniform size (205.1 ± 37.5 μm, *n* = 532). After 15 days of differentiation, con-EC/MC LOs smoothly differentiated into functional LOs and displayed higher ALB production and hepatic gene expression than ΔEC/MC LOs (deletion of ECs/MCs during LO generation) (Fig. 1d, e). This result indicates that the microenvironment provided by ECs and MCs promotes hepatic lineage differentiation in LOs. To investigate whether single donor-derived UC-ECs and UC-MCs also have the same roles in LO generation and differentiation, hiPSC-endoderm, UC-ECs, and UC-MCs were plated in the same culture system. Consistent with the results for con-EC/MC LOs, donor UC-EC/MC LOs with a small and uniform size (204.1 ± 35.5 μm, *n* = 527) were generated after 24 h of culture (Fig. 1c). Furthermore, ELISA analysis revealed that ALB production of UC-EC/MC LOs was equal to that of con-EC/MC LOs after 15 days of differentiation and significantly higher than that of ΔEC/MC LOs (Fig. 1d). qPCR analysis further confirmed that UC-EC/MC LOs displayed the same expression levels of the hepatic genes *ALB*, *CYP3A4*, glucose-6-phosphatase catalytic subunit (*G6PC*), and tryptophan 2,3-dioxygenase 2 (*TDO2*) as con-EC/MC LOs (Fig. 1e). These results indicate that single donor-derived UC-ECs and UC-MCs can promote LO differentiation as effectively as con-ECs and con-MCs and that single donor-derived UC-ECs and UC-MCs are promising cell sources for SDC-LO generation.

### Single donor-derived hiPSCs reprogrammed from UC-ECs

**Fig. 2:**
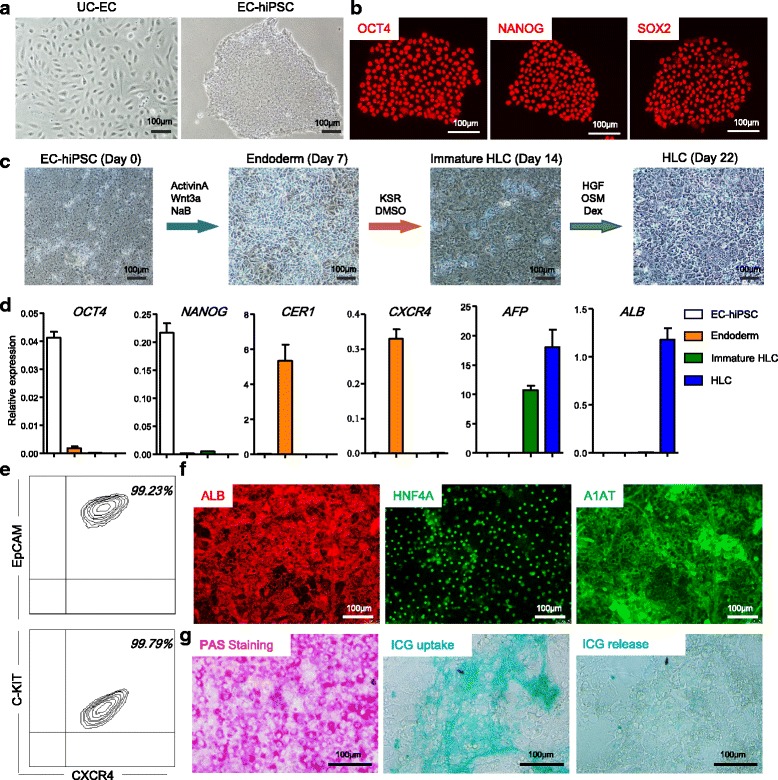
Single donor-derived hiPSCs reprogrammed from UC-ECs with efficient hepatic differentiation capacity. **a** Morphology of single donor-derived UC-ECs and EC-hiPSCs under phase-contrast microscopy. Scale bar, 100 μm. **b** Immunocytochemical detection of pluripotency factors OCT4, NANOG, and SOX2 in EC-hiPSCs. Scale bars, 100 μm. **c** Schematic illustration of the hepatic differentiation process and morphology of EC-hiPSCs, definitive endoderm, immature HLCs, and HLCs during differentiation. Scale bars, 100 μm. **d** Expression of pluripotency factors (*OCT4* and *NANOG*), definitive endoderm-related genes (*CER1* and CXCR4), an immature HLC-related gene (*AFP*), and an HLC-related gene (*ALB*) in EC-hiPSCs, definitive endoderm, immature HLCs, and HLCs, as determined by qPCR (*n* = 4 each). **e** Flow cytometry analysis of cells expressing EpCAM, CXCR4, and C-KIT among EC-hiPSC-derived definitive endoderm. **f** Immunocytochemical detection of the hepatic markers ALB, HNF4A, and A1AT in EC-hiPSC-derived HLCs. Scale bars, 100 μm. **g** Glycogen storage was detected by PAS staining (left), and ICG uptake and release (right) were analyzed in EC-hiPSC-derived HLCs. Scale bars, 100 μm. ALB albumin, EC endothelial cell, HGF hepatocyte growth factor, hiPSC human induced pluripotent stem cell, HLC hepatic-like cell, ICG indocyanine green, PAS periodic acid–Schiff, UC umbilical cord, OSM Oncostatin M, Dex Dexamethasone, KSR Knockout serum replacement, DMSO Dimethyl sulfoxide

In addition to the single donor-derived ECs and MCs, same donor-derived hiPSCs are also an indispensable component for SDC-LO generation. Human iPSCs have been reprogrammed successfully from different types of donor somatic cells, including fibroblasts, UC blood cells, and ECs [21]. The characteristics of UC-ECs, including their ease of collection from newborn tissue, few mutations, high efficiency of isolation and proliferation, and capacity for rapid reprogramming, make them an attractive somatic cell source for therapeutic-grade hiPSC generation [22, 23]. We transfected episomal hiPSC reprogramming vectors into UC-ECs using a method reported previously [18] to reprogram UC-ECs into hiPSCs (EC-hiPSCs) that exhibited morphologies typical of embryonic stem cells (Fig. 2a). The EC-hiPSCs were positive for nuclear expression of the embryonic stem cell markers OCT4, NANOG, and SOX2 (Fig. 2b). Furthermore, flow cytometry demonstrated that reprogrammed EC-hiPSCs had lost the expression of the endothelial marker CD31 while expressing typical hiPSC surface markers such as SSEA4 and TRA-1-60 (Additional file 3: Figure S2A). qPCR analysis further revealed that EC-hiPSCs expressed pluripotency-related genes such as *OCT4*, *NANOG*, *LIN28A*, *SOX2*, and *KLF4* at higher levels, exhibiting the downregulation of endothelial markers such as *CD31*, *TIE1*, *ERG*, and *vWF* compared to levels in the original UC-ECs (Additional file 3: Figure S2B).

### Effective differentiation of endoderm and hepatic lineages from single donor-derived EC-hiPSCs

To generate functional SDC-LOs, EC-hiPSCs should be able to differentiate into endoderm and hepatic lineages. We used a protocol reported previously to investigate hepatic lineage differentiation from these EC-hiPSCs (Fig. 2c) [7]. In endodermal differentiation medium, EC-hiPSCs showed rapid downregulation of the pluripotency genes *OCT4* and *NANOG* and upregulation of the endoderm-related genes *CER1* and *CXCR4* (Fig. 2c, d). Flow cytometry showed that the EC-hiPSC-endoderm was a highly pure population with 99.05 ± 0.26% CXCR4^+^EpCAM^+^ cells and 99.72 ± 0.10% CXCR4^+^C-KIT^+^ cells (Fig. 2e). Next, the endoderm culture medium was replaced with hepatic lineage differentiation medium as in Fig. 2c. EC-hiPSC-endoderm gradually differentiated into immature HLCs with high *AFP* and low *ALB* expression (Fig. 2c, d) and later matured into *ALB*-expressing HLCs (Fig. 2c, d). Immunocytochemical analysis showed that EC-hiPSC-derived HLCs were positive for ALB, HNF4A, and A1AT (Fig. 2f). Glycogen storage in these HLCs was demonstrated by PAS staining (Fig. 2g), and the capacity for ICG uptake and release was also confirmed (Fig. 2g). These results suggest that the single donor-derived EC-hiPSCs can be differentiated effectively into endoderm and hepatic cells and that the hiPSC-endoderm was a highly pure population, making it a promising endoderm source for SDC-LO generation.

### SDC-LO generation from single donor-derived hiPSC-endoderm, ECs, and MCs

**Fig. 3:**
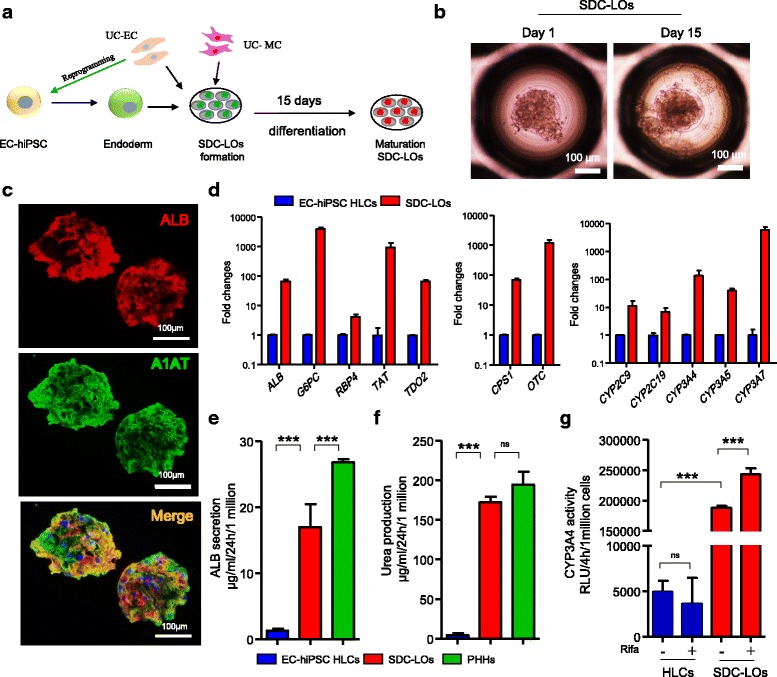
Single donor cell-derived LOs generated from single donor-derived hiPSC-endoderm, ECs, and MCs. **a** Schematic representation of the protocol for LO generation and differentiation from single donor-derived hiPSC-endoderm, ECs, and MCs. **b** Morphology of single donor cell-derived LOs (SDC-LOs) on days 1 and 15. Scale bars, 100 μm. **c** Immunocytochemical detection of ALB (red) and A1AT (green) expression in differentiated SDC-LOs on day 15. Scale bars, 100 μm. **d** Expression of common hepatic genes (*ALB*, *G6PC*, *RBP4*, *TAT*, and *TDO2*), urea/ammonia metabolism-related genes (*CPS1* and *OTC*), and cytochrome P450-encoding genes (*CYP2C9*, *CYP2C19*, *CYP3A4*, *CYP3A5*, and *CYP3A7*) in EC-iPSC-HLCs (blue, *n* = 6) and differentiated SDC-LOs (red, *n* = 6), determined by qPCR. **e** ALB production in EC-iPSC-HLCs (*n* = 6), differentiated SDC-LOs (*n* = 6), and primary human hepatocytes (PHHs, *n* = 4); data normalized to 1 million cells. **f** Urea production in EC-iPSC-HLCs (*n* = 3), differentiated SDC-LOs (*n* = 3), and PHHs (*n* = 4); data normalized to 1 million cells. **g** Quantification of CYP3A4 activity in EC-iPSC-HLCs (*n* = 4) and SDC-LOs (*n* = 4) with or without rifampicin (25 μM) induction; data normalized to 1 million cells. ***p < 0.001. ns not significant, ALB albumin, EC endothelial cell, HGF hepatocyte growth factor, hiPSC human induced pluripotent stem cell, HLC hepatic-like cell, LO liver organoid, MC mesenchymal cell, PHH primary human hepatocyte, UC umbilical cord, CYP cytochrome P450, G6PC glucose-6-phosphatase catalytic subunit, TDO2 tryptophan 2,3-dioxygenase

Combining the methods of cellular isolation, hiPSC reprogramming, and hepatic differentiation, we successfully obtained the essential cell sources for SDC-LO generation, including single donor-derived hiPSC-endoderm, ECs, and MCs with high purity. To investigate whether these cells can be used for SDC-LO generation, single donor-derived EC-iPSC-endoderm, UC-ECs, and UC-MCs were seeded simultaneously in a 3D microwell culture plate (Fig. 3a). The three cell types self-organized into LOs after 24 h of culture (Fig. 3b), and these SDC-LOs maintained an organoid morphology during extended culture (Fig. 3b). After 15 days of differentiation, immunocytochemical analysis revealed that SDC-LOs were successfully differentiated into functional hepatic lineages with positive expression of ALB and A1AT (Fig. 3c). Compared with EC-hiPSC-derived HLCs, differentiated SDC-LOs showed upregulation of the hepatic genes *ALB*, *G6PC*, *RBP4*, *TAT*, and *TDO2*; cytochrome P450-related genes *CYP2C9*, *CYP2C19*, *CYP3A4*, *CYP3A5*, and *CYP3A7*; and urea/ammonia metabolism-related genes *CPS1* and *OTC* (Fig. 3d). Moreover, the capacity for ALB secretion and urea production in the differentiated SDC-LOs was increased by 12.98-fold and 39.01-fold, respectively, compared to that in EC-iPSC-HLCs, reaching a level similar to that in PHHs (Fig. 3e, f). CYP3A4 activity in SDC-LOs was also 38.30-fold higher than that in EC-iPSC-HLCs, and CYP3A4 activity was induced by rifampicin in SDC-LOs but not in EC-iPSC-HLCs (Fig. 3g). These results indicate that single donor-derived hiPSC-endoderm, ECs, and MCs can be used for SDC-LO generation and that SDC-LOs can be effectively differentiated into functional organoids exhibiting ALB secretion and urea production abilities similar to those of PHHs.

### Functional SDC-LOs improve survival in ALF model mice

**Fig. 4:**
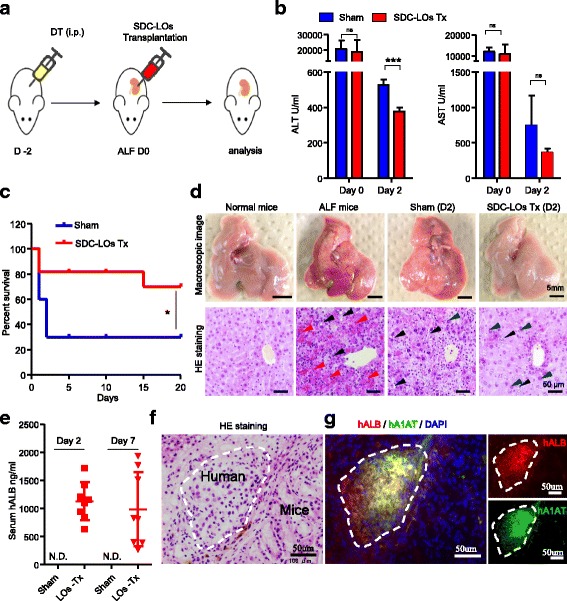
Single donor cell-derived LOs improve survival in mice with ALF. **a** Schematic representation of the protocol for generating ALF mice and transplanting SDC-LOs. **b** ALT and AST were detected on days 0 and 2 in the sham (*n* = 3) and SDC-LO-transplanted (*n* = 4) ALF mice. **c** Kaplan–Meier survival curves of ALF mice in the sham (*n* = 10) and SDC-LO-transplanted (*n* = 10) groups. **d** Macroscopic images (upper panel; scale bars, 5 mm) and hematoxylin and eosin (HE) staining (lower panel; scale bars, 50 μm) of the liver in normal mice, ALF mice, sham ALF mice, and SDC-LO-transplanted ALF mice on day 2. Red arrowhead, hepatocellular necrosis; black arrowhead, hepatocellular steatosis; blue arrowhead, enlarged hepatocyte. **e** Human-specific ALB was analyzed on days 2 and 7 in the sham (*n* = 3) and SDC-LO-transplanted (*n* = 8) groups. **f** HE staining of transplants on day 14. Scale bars, 50 μm. **g** Immunofluorescent analysis of human ALB and A1AT expression in transplants on day 14. Scale bars, 50 μm. *p < 0.05, ***p < 0.001. hALB human albumin, ALF acute liver failure, DT diphtheria toxin, ns not significant. N.D. not determined, SDC-LO single donor cell-derived liver organoid, Tx treatment, ALT alanine aminotransferase, AST aspartate aminotransferase

Next, we investigated whether the functional SDC-LOs could be used as an effective transplant in ALF therapy. We generated an ALF model in Alb-TRECK/SCID mice by intraperitoneal injection of DT (Fig. 4a) [20]. After 48 h, the mice developed a phenotype typical of ALF, as evidenced by increased serum ALT (> 15,000 IU/l) and AST (> 10,000 IU/l) levels (Fig. 4b), and 70% of the mice died within 7 days (Fig. 4c). Macroscopic images and histological analysis showed that the hepatocellular structure was damaged, and massive hepatocellular necrosis and hepatocellular steatosis were present with minimal inflammatory infiltration (Fig. 4d). After we transplanted differentiated SDC-LOs into the renal subcapsular spaces of ALF mice, the survival rate was significantly improved compared to that of the sham group (Fig. 4c). Indeed, 70% of mice showed recovery from ALF upon SDC-LO transplantation (Fig. 4c), with decreased serum ALT and AST levels at 2 days after transplantation (Fig. 4b). We also observed a decrease in serum ALT and AST levels in the sham group, but the level of ALT was still higher than that in LO-transplanted mice (Fig. 4b). In SDC-LO-transplanted ALF mice, the injured liver underwent a regeneration process with enlarged hepatocytes and an increase in hepatic nuclear size (Fig. 4d) [24]. To further confirm that transplanted SDC-LOs can perform hepatic functions in ALF mice, a human-specific hepatic protein, human ALB, was measured in transplanted mice by ELISA. In SDC-LO-transplanted mice, human ALB was detected at concentrations of 1128 ± 338.1 ng/ml and 988.2 ± 660.3 ng/ml at 2 days and 7 days after transplantation, respectively (Fig. 4e). After 2 weeks, the kidneys of mice transplanted with SDC-LOs were collected, and histological analysis revealed that the transplants had hepatic cord-like structures characteristic of the adult liver (Fig. 4f) and were comprised of cells expressing human-specific ALB and A1AT (Fig. 4g). These results indicate that differentiated SDC-LOs were able to rapidly perform hepatic functions in ALF mice and improve the microenvironment for liver regeneration to promote recovery from ALF.

## Discussion

To ensure that LOs can be produced on a large enough scale for further clinical application, the three required cell types should be derived from a single donor and capable of large-scale expansion or production in vitro. ECs and hiPSCs can be stably expanded in vitro, whereas BM-MCs exhibit a low proliferative capacity that is gradually lost over repeated passaging [25]. In this study, we found that UC-MCs represented a suitable replacement for BM-MCs in LO generation and differentiation. Compared with BM-MCs, UC-MCs have a higher proliferation rate and can be isolated from newborns without invasive procedures [25]; these characteristics of UC-MCs provide a special advantage for massively generating LOs and enabling the application of LOs in regenerative medicine. Although some differences between UC-MCs and BM-MCs have been reported [26], UC-MCs could serve the same function as BM-MCs in LO generation and differentiation when combined with ECs. More importantly, the use of UC-MCs makes it possible to generate LOs from a single donor.

Liver transplantation is the only proven therapy for ALF but is limited by the shortage of HLA-matched donor livers. The risk of death increases without HLA matching in liver transplantation [2, 4]. Host cell-derived or HLA-matched donor-derived transplants have traditionally been used to avoid immune rejection. The use of hiPSCs reprogrammed from host somatic cells has led to unprecedented opportunities for generating host-derived or HLA-matched donor-derived cells and organoids [27]. Transplantation of HLA-matched iPSC-derived epithelial cells and neurons in recipients resulted in attenuation of the immune response, with normal function of transplanted cells [28, 29]. In this study, we confirmed that functional LOs can be generated from single donor-derived cells and improve the survival rate of ALF mice after transplantation, underscoring the possibility of using self-derived LOs or HLA-matched LOs for liver disease therapy. The use of homozygous HLA LOs is thought to exhibit potential for further clinical application of an effective LO banking system [30]. Previous studies have estimated that a biobank of hiPSCs from 150 selected homozygous HLA-typed volunteers would match 93% of the population in the United Kingdom [31], and 50 selected homozygous HLA donors would cover 90.7% of the Japanese population [32]. By collecting single donor-derived ECs and MCs from such selected homozygous HLA-typed donors and generating same donor-derived hiPSCs, an LO biobank including homozygous HLA-typed hiPSCs, ECs, and MCs from the same donor could be established that could provide the majority of the population with LOs for ALF treatment as well as the treatment of other liver diseases.

The liver is the major organ involved in the detoxification of various metabolites. In ALF patients, the rapid loss of liver metabolic function results in the accumulation of toxic metabolites that cause further injury to the body [1]. ALF is a severe form of liver injury that is potentially reversible, as the rapid elimination of toxic metabolites can promote liver regeneration and minimize the risk of post-transplant complications [1]. To eliminate toxic metabolites, a bioartificial liver support system has been developed based on induced human functional hepatocytes that can modulate blood levels of ammonia and bilirubin, prolonging survival in a porcine ALF model [33]. Here, we found that transplanted SDC-LOs could rapidly perform hepatic functions and that the ALT levels in LO-transplanted mice decreased faster decrease those in the sham group. These findings indicate that SDC-LOs perform metabolic detoxification and synthetic functions in ALF mice and eliminate the accumulation of toxic metabolites to provide a healthy microenvironment for liver regeneration, thereby improving survival. The ability of SDC-LOs to quickly perform hepatic functions may be attributable to the culture system. SDC-LOs were generated and differentiated in a 3D suspension microwell plate that enabled the collection of LOs without enzyme treatment and maximally preserved LO function for transplantation. This simple collection method also enables the collection of functional LOs produced on a large scale in a short amount of time for clinical transplantation.

## Conclusions

In this study, we successfully obtained single donor-derived hiPSC-endoderm, ECs, and MCs with high purity. These three type cells were able to organize into SDC-LOs and differentiate into functional organoids that performed hepatic functions in ALF mice and improved the survival rate. Although further efforts are necessary to evaluate the use of SDC-LOs in clinical treatment, this proof-of-concept demonstration of functional SDC-LO transplantation provides a promising novel approach for ALF therapy.

## Additional files

Additional file 1: Table S1. Presenting a list of quantitative RT-PCR primers and probes for human genes. (XLSX 10 kb)

Additional file 2: Figure S1. Showing isolation and characterization of endothelial cells (ECs) and mesenchymal cells (MCs) from a single human umbilical cord (UC). **A** Schematic representation of the process of EC and MC isolation from human UC. **B** Morphology of UC-derived ECs (passage 1) and MCs (passage 2). **C** Images of the capillary vascular network formation by con-ECs and UC-ECs on Matrigel matrix. **D** Proliferation rate of con-ECs (blue, *n* = 4) and UC-ECs (red, *n* = 4) after 4 days of culture. **E** Proliferation rate of con-MCs (blue) and UC-MCs (red) after 4 days of culture. **F** Flow cytometry analysis of con-EC (one lot) and UC-EC (three donors) surface markers: CD31, CD144, CD146, CD90, CD45, and SSEA4. **G** Flow cytometry analysis of con-MC (one lot) and UC-MC (three donors) surface markers: CD90, CD73, CD105, CD31, CD45, and HLA-DR. (PDF 477 kb)

Additional file 3: Figure S2. Showing characterization of hiPSCs reprogrammed from human UC-ECs. **A** Flow cytometry analysis of UC-ECs and EC-iPSCs expressing TRA-1-60, SSEA4, and CD31. **B** Expression of pluripotency-related genes (*OCT4*, *NANOG*, *LIN28A*, *SOX2*, and *KLF4*) and EC-related genes (*CD31*, *TIE1*, *ERG*, and *vWF*) in UC-ECs (*n* = 4) and EC-hiPSCs (*n* = 4), as determined by qPCR (*n* = 4). (PDF 152 kb)

## Abbreviations

3D: Three-dimensional
ALB: Albumin
ALF: Acute liver failure
ALT: Alanine aminotransferase
AST: Aspartate aminotransferase
bFGF: Basic fibroblast growth factor
BM: Bone marrow
CD: Cluster of differentiation
CYP: Cytochrome P450
DT: Diphtheria toxin
EC: Endothelial cell
EGF: Epidermal growth factor
EGM: Endothelial cell growth medium
ELISA: Enzyme-linked immunosorbent assay
G6PC: Glucose-6-phosphatase catalytic subunit
HGF: Hepatocyte growth factor
hiPSC: Human induced pluripotent stem cell
HLA: Human leukocyte antigen
HLC: Hepatic-like cell
LO: Liver organoid
MC: Mesenchymal cell
MSCGM: Mesenchymal stem cell growth medium
qPCR: Quantitative polymerase chain reaction
SDC-LO: Single donor cell-derived liver organoid
SFD: Serum-free differentiation
TDO2: Tryptophan 2,3-dioxygenase
UC: Umbilical cord
VEGF: Vascular endothelial growth factor
