# Supplementary figures and images for: Human liver organoids generated with single donor-derived multiple cells rescue mice from acute liver failure
Source: Stem Cell Res Ther. 2018 Jan 10;9:5. doi: 10.1186/s13287-017-0749-1 (PMC5763644; doi:10.1186/s13287-017-0749-1)

Figure S1. Isolation and characterization of ECs and MCs from a single human UC.

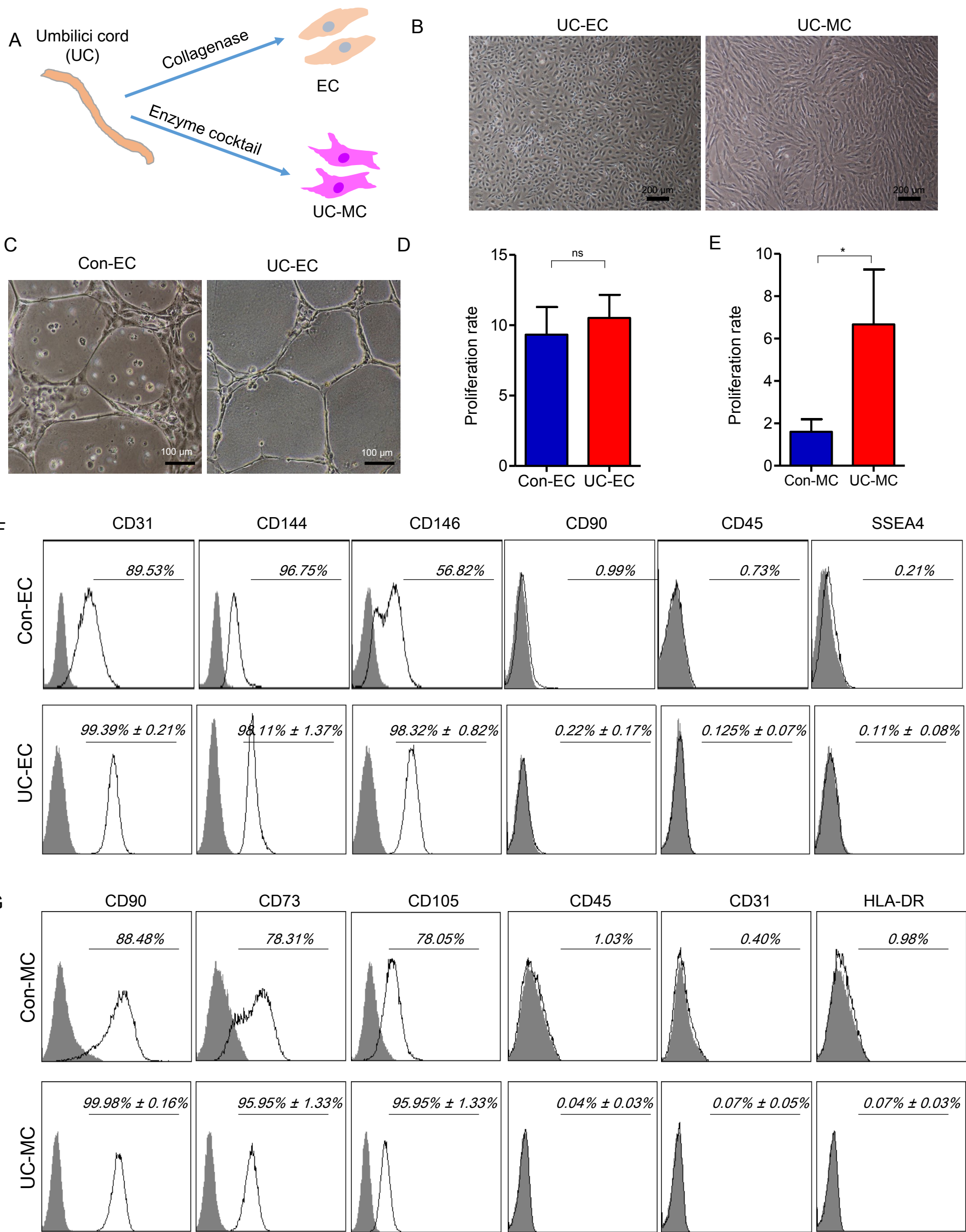

Supplement: Supplementary file 2 — Showing isolation and characterization of endothelial cells (ECs) and mesenchymal cells (MCs) from a single human umbilical cord (UC). A Schematic representation of the process of EC and MC isolation from human UC. B Morphology of UC-derived ECs (passage 1) and MCs (passage 2). C Images of the capillary vascular network formation by con-ECs and UC-ECs on Matrigel matrix. D Proliferation rate of con-ECs (blue, n = 4) and UC-ECs (red, n = 4) after 4 days of culture. E Proliferation rate of con-MCs (blue) and UC-MCs (red) after 4 days of culture. F Flow cytometry analysis of con-EC (one lot) and UC-EC (three donors) surface markers: CD31, CD144, CD146, CD90, CD45, and SSEA4. G Flow cytometry analysis of con-MC (one lot) and UC-MC (three donors) surface markers: CD90, CD73, CD105, CD31, CD45, and HLA-DR. (PDF 477 kb) [file 13287_2017_749_MOESM2_ESM.pdf]

Figure S2 Characterization of hiPSCs reprogramed from human UC-ECs.

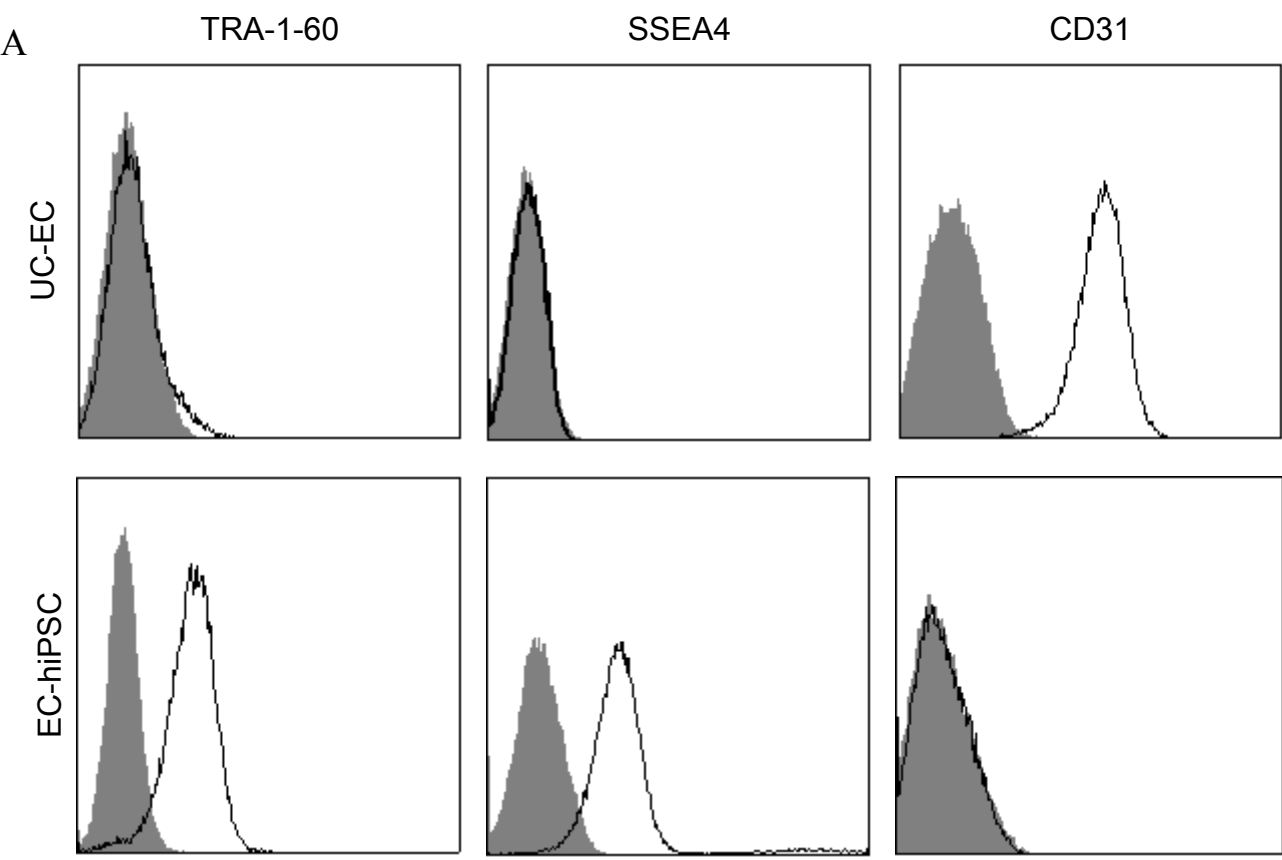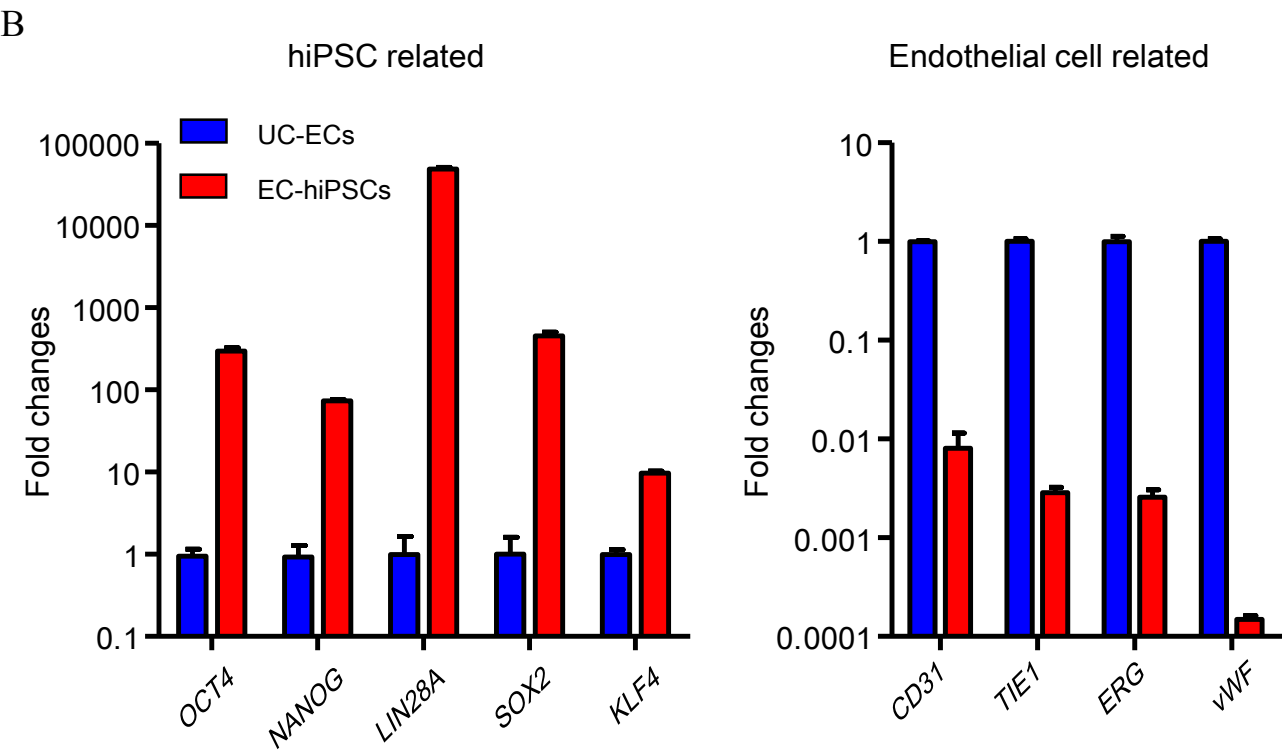

Supplement: Supplementary file 3 — Showing characterization of hiPSCs reprogrammed from human UC-ECs. A Flow cytometry analysis of UC-ECs and EC-iPSCs expressing TRA-1-60, SSEA4, and CD31. B Expression of pluripotency-related genes (OCT4, NANOG, LIN28A, SOX2, and KLF4) and EC-related genes (CD31, TIE1, ERG, and vWF) in UC-ECs (n = 4) and EC-hiPSCs (n = 4), as determined by qPCR (n = 4). (PDF 152 kb) [file 13287_2017_749_MOESM3_ESM.pdf]
